# Supplementary material for: Role of Crystalline Si and SiC Species in the Performance of Reduced Hybrid C/Si Gels as Anodes for Lithium-Ion Batteries
Source: Nanomaterials (Basel). 2023 Jan 23;13(3):458. doi: 10.3390/nano13030458 (PMC9919348; doi:10.3390/nano13030458)
Supplement: Supplementary file 1 [file nanomaterials-13-00458-s001.zip › Supplementary nanomaterials.pdf]

Developing *c*Si and SiC species in polymeric materials for the enhancement of lithium-ion batteries performance

Samantha L. Flores-López, Belén Lobato, Natalia Rey-Raap, Ignacio Cameán, Ana B. García, Ana Arenillas

[1]Instituto de Ciencia y Tecnología del Carbono, INCAR-CSIC. Francisco Pintado Fe, 26. 33011 Oviedo, Spain

*corresponding author:

SUPPLEMENTARY MATERIAL

1. Porosity calculation

Equation to calculate porosity percentage from envelop density, obtained with Geopyc analyzer, from Micromeritics, and helium density:

$$P = \left(1 - \frac{\rho_{Env}}{\rho_{He}}\right) 100 \qquad \text{[Equation 1]}$$

2. Optimization of active mass of the electrodes

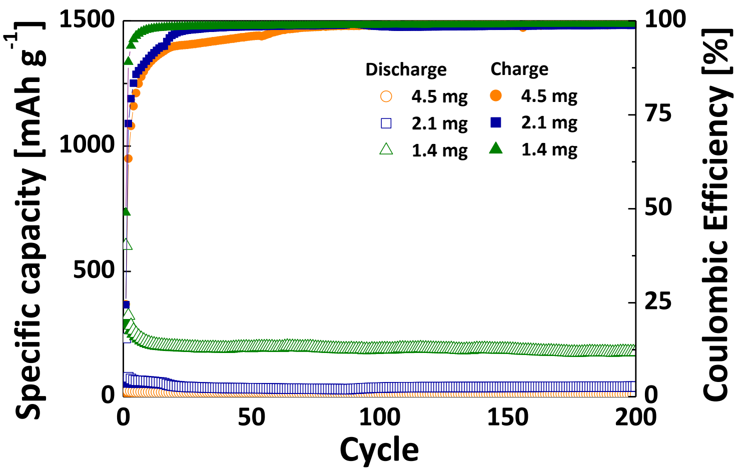

Figure S1. Specific capacity and coulombic efficiency for sample rHG/85-1, using electrodes with different active mass from the galvanostatic cycling at 1000 mA g^{-1}.

3. Complementary XPS analysis

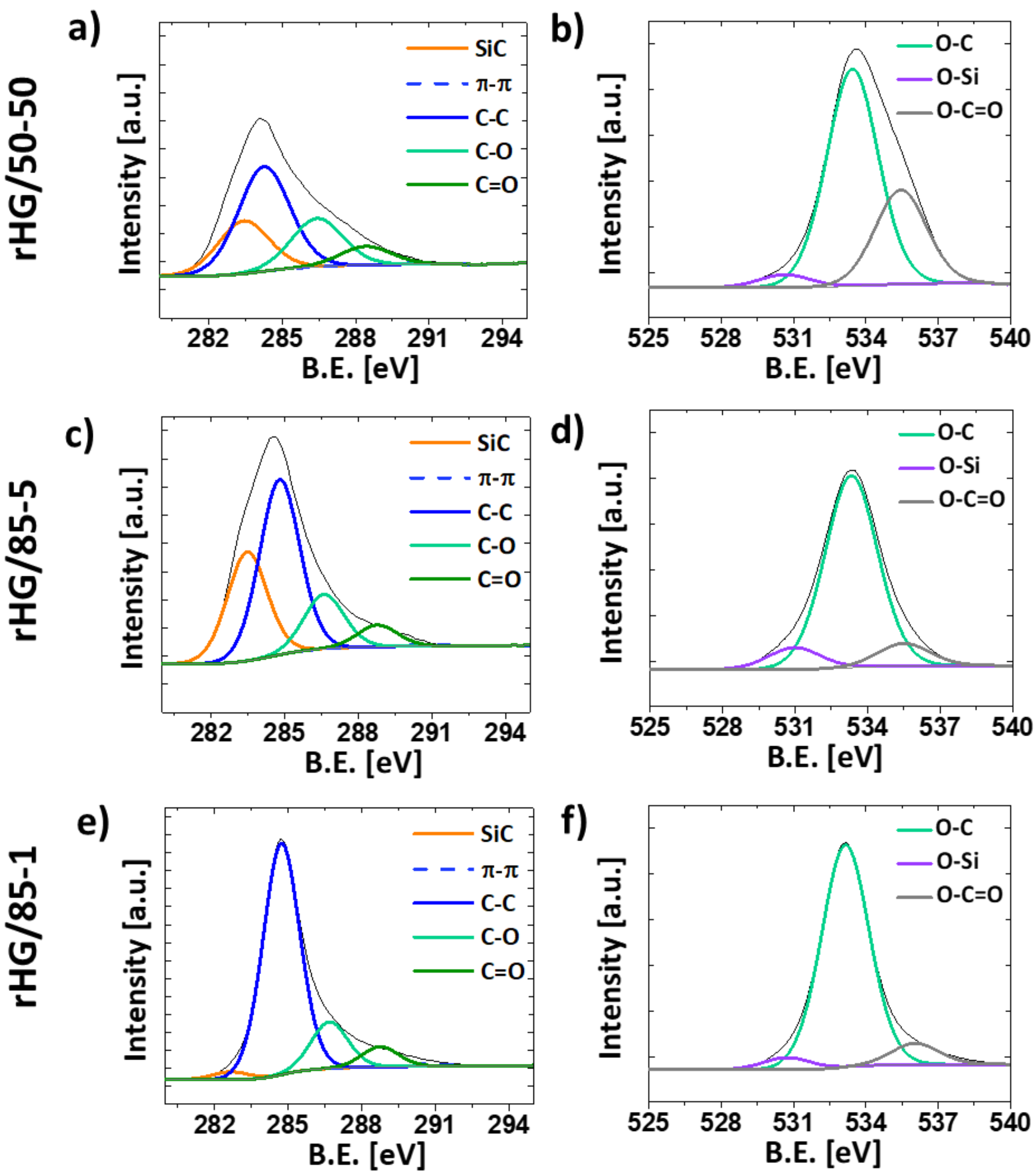

Figure S2a. Deconvolution of the high-resolution XPS spectra of the C 1 s region (a, c, e) and O 1s region (b, d, f) for some reduced C-Si hybrid gels.

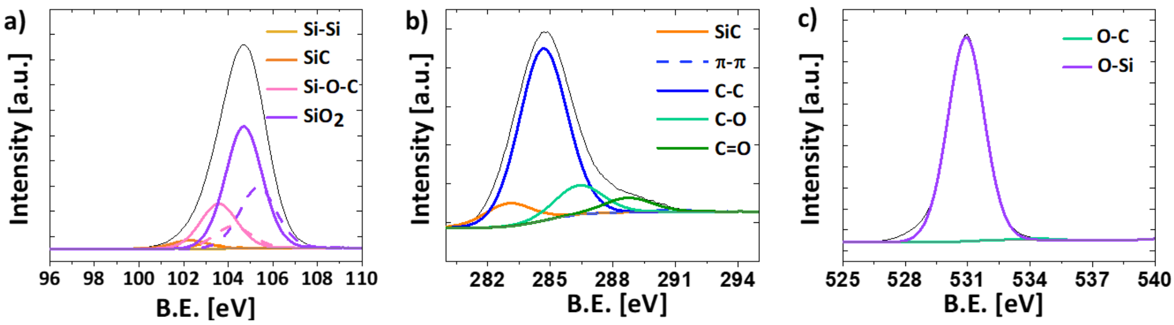

Figure S2b. Deconvolution of the high-resolution XPS spectra of the Si 2p region (a), C 1 s region (b) and O 1s region (c) for HG/85 hybrid gel.

4. Complementary XRD analysis

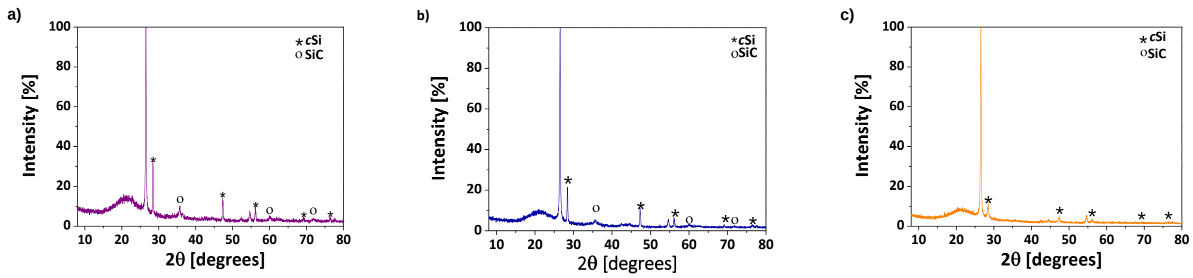

Figure S3. XRD patterns of rHG/85 reduced with different heating rates, using commercial graphite as reference a) rHG/85-50, b) rHG/85-5 and c) rHG/85-1

5. Reduced composite rCG/85-5

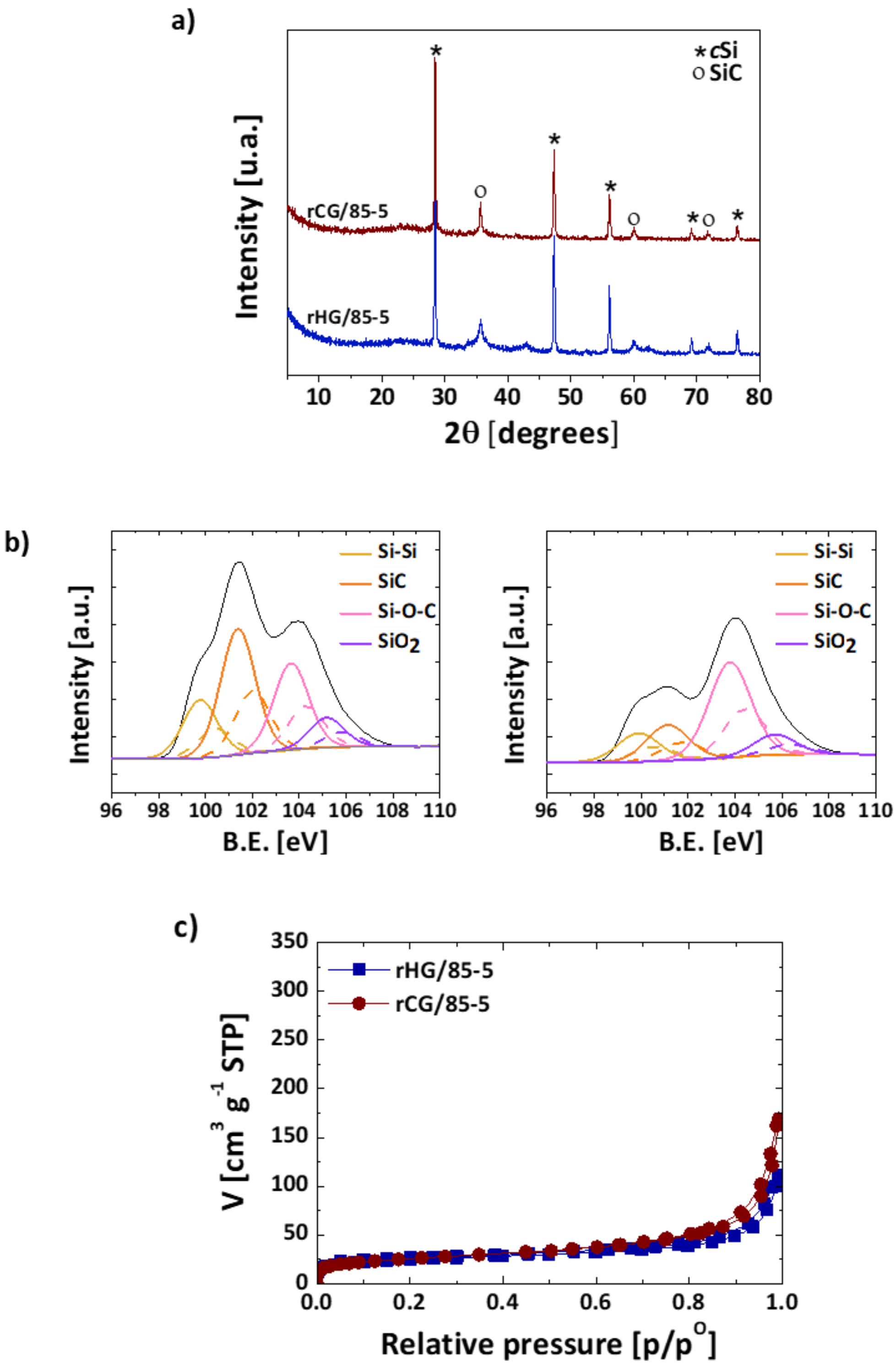

Figure S4. Comparative of a) crystalline species (by XRD diffraction), b) silicon distribution (by XPS spectra) and c) porous properties (by N_2 adsorption-desorption isotherms at 77 K) between the reduced C-Si hybrid (rHG/85-5) and composite (rCG/85-5).

6. Comparison with commercial Graphite

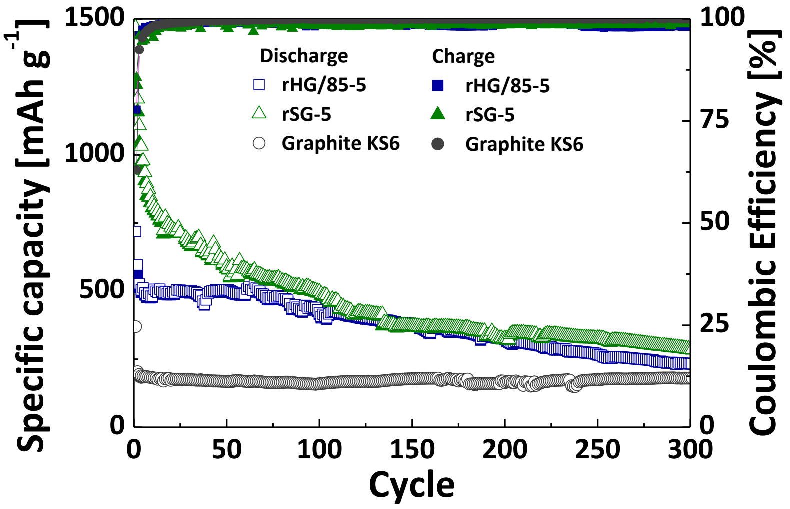

Figure S5. Galvanostatic performance of rSG-5, rHG/85-5 and commercial graphite Timrex KS6 measured at 500 mA g^{-1}.

7. Effect of precoating

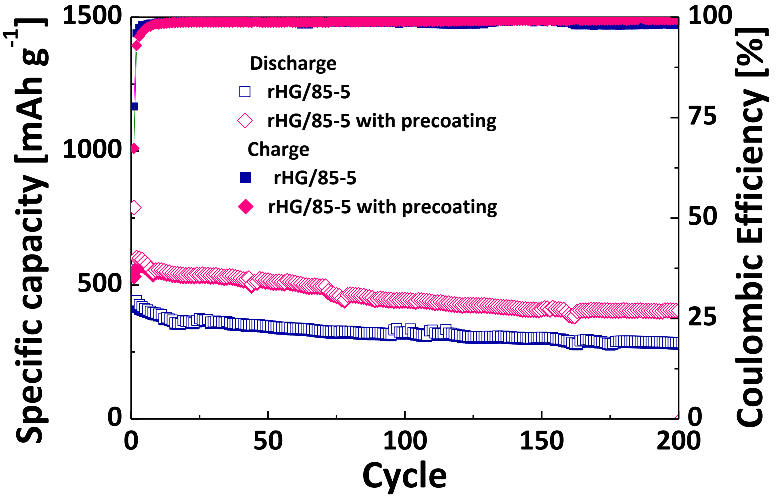

Figure S6. Effect of precoating the electrode support with a NaCMC-C65 layer on galvanostatic cycling at 500 mA g^{-1}.
